# Performance verification of anti-SARS-CoV-2-specific antibody detection by using four chemiluminescence immunoassay systems

**DOI:** 10.1177/0004563220963847

**Published:** 2020-11-12

**Authors:** Yafang Wan, Zhijie Li, Kun Wang, Tian Li, Pu Liao

**Affiliations:** Department of Clinical Laboratory, Chongqing General Hospital, Chongqing, China

**Keywords:** COVID-19, SARS-CoV-2, antibody, chemiluminescence immunoassay, performance verification

## Abstract

**Objectives:**

The purpose of the current study was to evaluate the analytical performance of seven kits for detecting IgM/IgG antibodies against coronavirus (SARS-CoV-2) by using four chemiluminescence immunoassay systems.

**Methods:**

Fifty patients diagnosed with SARS-CoV-2 infection and 130 controls without coronavirus infection from the General Hospital of Chongqing were enrolled in the current retrospective study. Four chemiluminescence immunoassay systems, including seven IgM/IgG antibody detection kits for SARS-CoV-2 (A_IgM, A_IgG, B_IgM, B_IgG, C_IgM, C_IgG and D_Ab), were employed to detect antibody concentrations. The chi-square test, the receiver operating characteristic (ROC) curve and Youden’s index were determined to verify the cut-off value of each detection system.

**Results:**

The repeatability verification results of the A, B, C and D systems are all qualified. D_Ab performed best (92% sensitivity and 99.23% specificity), and B_IgM performed worse than the other systems. Except for the A_IgM and C_IgG systems, the optimal diagnostic thresholds and cut-off values of the other kits and their recommendations are inconsistent with each other. B_IgM had the worst AUC, and C_IgG had the best diagnostic accuracy. More importantly, the B_IgG system had the highest false-positive rate for testing patients with AIDS, tumours and pregnancies. The A_IgM system test showed the highest false-positive rates among elderly individuals over 90 years old. COVID-2019 IgM/IgG antibody test systems exhibit performance differences.

**Conclusions:**

The Innodx Biotech Total Antibody serum diagnosis kit is the most reliable detection system for anti-SARS-CoV-2 antibodies, which can be used together with nucleic acid tests as an alternative method for SARS-CoV-2 detecting.

## Introduction

Coronavirus pneumonia (coronavirus disease 2019, COVID-19) is an acute respiratory infection caused by severe acute respiratory syndrome coronavirus type 2 (SARS-CoV-2).^1^ The epidemic of the disease has not ended since the winter of 2019, and it is still raging worldwide. SARS-CoV-2 is highly contagious through aerosols, droplets and contact.^2^ Generally, the incubation period of SARS-CoV-2 is three to seven days, but the longest incubation period can reach 14 days.^3^ It has caused more than 7,250,000 human infections and nearly 410,000 deaths worldwide as of the end of 9 June. Therefore, the early diagnosis of SARS-CoV-2 infection is crucial. Previous studies have shown that the SARS-CoV-2 antigen stimulates the immune system to produce an immune response and that specific IgM and IgG antibodies appear in the serum of patients after infection.^4^ The SARS-CoV-2-specific IgM and IgG antibody tests have been involved in the diagnosis criteria for suspected patients whose COVID-19 viral nucleic acid test appears false negative, according to the recently published guidelines of Novel Coronavirus Pneumonia Diagnosis and Treatment (Trial Version 7), which were advocated by the National Health Committee.^5^

Current popular detection methods for anti-SARS-CoV-2 antibodies include colloidal gold and chemiluminescence immunoassays.^6^ Chemiluminescence immunoassays are a laboratory technology that combines a luminescence system with an immune response. It not only uses the specificity of the immune response but also has the high sensitivity of the luminescence reaction and is widely used in immunoassays.^7^ Our laboratory currently has four automatic chemiluminescence immunoassay systems, A, B, C and D, of which the three detection systems A, B and C detect SARS-CoV-2-specific IgM and IgG antibodies, and the D system detects total IgM/IgG antibodies. The current investigation intends to evaluate the repeatability, clinical sensitivity and specificity of seven antibody detection kits for four detection systems, as well as the false-positive rate in special populations. Youden’s index verifies the best diagnostic threshold (cut-off value) of each detection system to understand the analytical detection performance of each system and ensure the detection results.

## Material and methods

### Sample collection

Fifty serum samples from patients with SARS-CoV-2 infection diagnosed in 26 January to 6 February 2020 and 130 serum samples from patients with other conditions, including 20 late-term pregnant women, 20 patients with solid tumours, 20 patients with AIDS, 21 patients over 90 years old and 49 normal controls, were enrolled from the Immunology Department of the Laboratory Department of Chongqing General Hospital (three hospitals) from late February to March 2020. Control populations are selected based on common false-positive populations (interfering factors, such as rheumatoid factor, heterophilic antibody, complement, acquired animal Ig antibody, lysozyme, etc.) reported in the daily work and literature reports. All patients with SARS-CoV-2 infection were confirmed by nucleic acid testing (NAT) and computed tomography (CT) scan. All collected serum specimens were inactivated in a water bath at 56°C for 1 h and then stored in a freezer at –80°C.^8,9^

### Reagents and instruments

The automatic immunochemiluminescence analyser A was called detection system A (Bioscience Diagnostic Technology Co., Ltd). Reagents included the anti-coronavirus (SARS-CoV-2) IgM antibody detection kit (referred to as A_IgM, batch number: G202002415), and S/CO (sample cut-off value) ≥1.0 was designated positive. For the SARS-CoV-2 IgG antibody detection kit (referred to as A_IgG, batch number: G202002414), S/CO ≥ 1.0 was designated positive. The fully automatic immunochemiluminescence analyser B was called detection system B (Shenzhen New Industries Biomedical Engineering Co., Ltd). Reagents included the SARS-CoV-2 IgM antibody detection kit (referred to as B_IgM, batch number: 271200201), for which S/CO ≥ 1.0 AU/ml was designated positive. For the SARS-CoV-2 IgG antibody detection kit (referred to as B_IgG, batch number: 2722000101), S/CO ≥ 1.0 AU/ml was designated positive. The automatic immunochemiluminescence analyser C was called detection system C (Shenzhen YHLO Biotech Co., Ltd). Reagents included the severe acute respiratory syndrome coronavirus 2 (SARS-CoV-2) IgM antibody detection kit (referred to as C_IgM, batch number: 20200206), for which S/CO ≥ 10 AU/ml was designated positive. For the SARS-CoV-2 IgG antibody detection kit (referred to as C_IgG, batch number: 20200202), S/CO ≥ 10 AU/ml was designated positive. The fully automatic immunochemiluminescence analyser D was called detection system D (Xiamen Innodx Biotech Co., Ltd). Reagents included the SARS-CoV-2 antibody detection kit (referred to as D_Ab, batch number: 20200309), for which S/CO ≥ 1.0 was designated positive.

### Precision verification

Under the conditions of calibration and quality control of the detection systems, all of them are qualified, and the following experiments are carried out. The cut-off value is 1.0, 1.0, 1.10 AU/ml, 1.10 AU/ml, 10 AU/ml, 10 AU/ml and 1.0 in the A_IgM, A_IgG, B_IgM, B_IgG, C_IgM, C_IgG and D_Ab detection systems, respectively. Among 50 specimens of patients infected with SARS-CoV-2, one case of a weakly positive specimen with an S/CO value less than three times the cut-off value (level 1, L1) and one case with an S/CO value greater than three times the cut-off value (level 2, L2) were selected. Within-run precision was determined first. All detection systems were used to analyse the corresponding L1 and L2 specimens, conducting 20 consecutive tests. All tests were completed within one day, 20 S/CO values were observed, the results were judged and the standard deviation and coefficient of variation were calculated. The result was judged to be 100% in line, and the coefficient of variation was less than 10%. Between-run precision was examined second. The detection system analyses the corresponding L1 and L2 specimens, which were performed once per day, continuously detecting for 20 days, observing the S/CO value 20 times, judging the result and calculating the standard deviation and coefficient of variation. The result was judged to be 100% in line, and the coefficient of variation was less than 15%.

### Statistical analysis

All statistical analyses were conducted using R software (http://www.R-project.org/).

Evaluations of sensitivity with the 95% CI, specificity with the 95% CI and false positives in specific populations were conducted separately. The ROC curve (R package pROC) and Youden’s index were used to calculate the optimal diagnostic threshold (cut-off value) of the detection system.

## Results

Clinical characteristics of all samples are present in Table 1. Sex distribution between two groups shows no significant difference. Age in case group was younger than control groups. About 32% patients endure severe respiratory syndrome.

**Table 1.** Clinical characteristic of all samples.

Characteristics	Case (*n* = 50)	Control (*n* = 130)
Sex（male/female）	22/28	70/60
Age (mean ± SD)	47.44 ± 17.0	61.9 ± 23.0
Symptomatic		
Severe	16 (32%)	
Mild	34 (68%)	
Complications (none/Yes)		
Cough	15/35	
Fever	15/35	
Fatigue	39/11	
Dyspnoea	40/10	

To test the precision of each kit, we performed within-run and between-run detections. As seen from Table 2, the repeatability verification results of the A, B, C and D systems are all qualified. Among them, system D performed best and system B performed worst in the weak-positive specimens. More importantly, the B_IgM and B_IgG systems were nearly twice as precise as C_IgM and D_Ab.

**Table 2.** Diagnosis precision within different kits.

Kits	Within-run		Between-run	
	L1 (CV%)	L2 (CV%)	L1 (CV%)	L2 (CV%)
A_IgM	2.9 ± 0.17 (5.71)	357.4 ± 11.95 (3.34)	2.85 ± 0.22 (7.72)	349.9 ± 12.76 (3.64)
A_IgG	1.51 ± 0.06 (4.19)	205.3 ± 7.59 (3.7)	1.66 ± 0.10 (6.02)	208.6 ± 8.22 (3.94)
B_IgM	1.32 ± 0.04 (3.18)	3.74 ± 0.34 (8.98)	1.45 ± 0.07 (4.83)	3.88 ± 0.45 (11.6)
B_IgG	1.94 ± 0.16 (8.04)	22.75 ± 0.61 (2.7)	2.11 ± 0.19 (9)	21.09 ± 0.63 (3)
C_IgM	32.7 ± 0.92 (2.8)	163.9 ± 4.27 (2.6)	35.7 ± 1.13 (3.12)	160.5 ± 4.87 (3.03)
C_IgG	16.11 ± 0.69 (4.25)	117.43 ± 1.50 (1.28)	17.0 ± 0.74 (4.35)	115.1 ± 1.55 (1.35)
D_Ab	2.74 ± 0.06 (2.15)	23.05 ± 0.57 (2.46)	2.88 ± 0.09 (3.13)	23.67 ± 0.59 (2.49)

L1: level 1; L2: level 2.

A total of 50 patients were considered to have COVID-19 because a viral nucleic acid test appeared positive, and the other 130 controls had negative viral nucleic acid results. Overall, 180 subjects were tested with the COVID-19-specific serological assay. The results showed varying sensitivity and specificity among different kits. D_Ab performed best (92% sensitivity and 99.23% specificity), and B_IgM performed worse than the others (Table 3).

**Table 3.** Diagnosis sensitivity and specificity within different kits.

Kits	Positive in case	Positive in control	Sensitivity (95%CI)	Specificity (95%CI)
A_IgM	41 (9)	8 (122)	82.00% (69.20%,90.23%)	93.85% (88.33%,96.85%)
A_IgG	43 (7)	4 (126)	86.00% (73.81%,93.05%)	96.92% (92.36%,98.80%)
B_IgM	13 (37)	8 (122)	26.00% (15.87%,39.55%)	93.85% (88.33%,96.85%)
B_IgG	43 (7)	28 (102)	86.00% (73.81%,93.05%)	78.46% (70.63%,84.66%)
C_IgM	31 (19)	3 (127)	62.00% (48.15%,74.14%)	97.69% (93.44%,99.21%)
C_IgG	44 (6)	3 (127)	88.00% (76.20%,94.38%)	97.69% (93.44%,99.21%)
D_Ab	46 (4)	1 (129)	92.00% (81.16%,96.85%)	99.23% (95.77%,99.86%)

**Figure 1.**
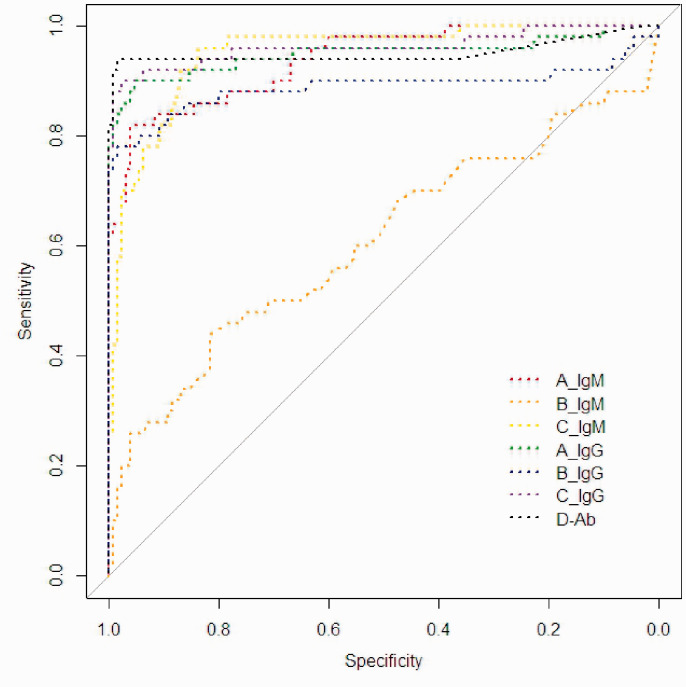
ROC curve for different kits.

The ROC curve was depicted by using the original S/CO value (Figure 1). According to the ROC curve, we obtained the optimal operating point of the different kits (Table 3). It can be concluded that, except for the optimal operating thresholds of A_IgM and C_IgG, the optimal diagnostic thresholds of the other kits and the cut-off values from the recommendations are inconsistent with each other. The results showed that the AUC of D_Ab reached 0.95 and that Youden’s index was 0.93 (Table 4). The optimal cut-off value was 0.54, with sensitivity and specificity values of 99% and 94%, respectively. According to the optimal operating threshold, there were only three patients who had a negative result, and two controls had a positive result. Additionally, B_IgM had the worst AUC, and C_IgG had the best diagnostic accuracy.

**Table 4.** Cut-off value and ROC-related parameters within different kits.

Kit	Cut-off value	Optimal operating point	Specificity	Sensitivity	AUC	Youden’s index
A_IgM	1	0.9	0.96	0.82	0.94	0.78
A_IgG	1	0.49	0.95	0.9	0.95	0.85
B_IgM	0.9	0.56	0.82	0.44	0.61	0.26
B_IgG	0.9	3.17	0.99	0.78	0.89	0.77
C_IgM	10	1.85	0.84	0.96	0.95	0.8
C_IgG	10	9.61	0.98	0.9	0.96	0.88
D_Ab	1	0.54	0.99	0.94	0.95	0.93

Considering that endogenous and exogenous factors exist in the process of antibody assays, subgroups of controls, including patients with acquired immune deficiency syndrome (AIDS), tumours, or pregnancies or those older than 90 years old, were involved in the current analysis. Each system had false-positive results in the selected subgroup of controls (Table 5). It is worth noting that the B_IgG system had the highest false-positive rate for testing patients with AIDS, tumours and pregnancies. The A_IgM system test showed the highest false-positive rates among elderly individuals over 90 years old.

**Table 5.** The false-positive rate (%) in specific patients.

Subgroup	A_IgM	A_IgG	B_IgM	B_IgG	C_IgM	C_IgG	D_Ab
HIVs (*n* = 20)	5	0	5	55	5	5	5
Tumours (*n* = 20)	10	15	10	40	5	0	0
Pregnant (*n* = 20)	5	0	10	15	0	0	0
Elder (≥90, *n* = 21)	9.5	0	4.8	0	4.8	4.8	0

## Discussion

SARS-CoV-2 belongs to the β genus and is the seventh most well-known coronavirus that infects humans. Its nucleocapsid protein (NP) stimulates the human immune system to cause chemical reactions. Specific IgM antibodies emerge on the seventh day of infection and appear to peak after 28 days. Specific IgG antibodies emerge around the 10th day of infection and reach a peak after 49 days, which can be maintained for a long time in the blood. The median time for total plasma antibodies appears on the 12th day after infection.^10,11^ In the current investigation, the average time of serum collection in all subjects was 13 days after diagnosis; therefore, specific IgM and IgG antibodies should already exist in the specimens.

With the published guidelines of the Novel Coronavirus Pneumonia Diagnosis and Treatment Program (Trial Version 7),^5^ the suspected cases are positive for serum SARS-CoV-2-specific IgM/IgG antibodies, are positive for SARS-CoV-2-specific IgG antibodies, or have SARS-CoV-2 IgG antibody concentrations that are four times greater in the recovery period than in the acute period, which can confirm the diagnosis of COVID-19.^12,13^ The diagnosis standard of COVID-19 is a changing situation. There is substantial market demand for SARS-CoV-2 antibody detection reagents worldwide. Manufacturers domestically produce antibody detection reagents that are used in the clinical laboratory. Previous investigations have shown that the clinical specificity and sensitivity of some anti-SARS-CoV-2 IgM antibodies are 96.2% and 70.24%, respectively. The clinical specificity and sensitivity of anti-SARS-CoV-2 IgG antibodies are 92.4% and 96.1%, respectively. Therefore, false-negative and false-positive results will appear, which will cause confusion in clinical judgment. Thus, the laboratory needs to pay close attention to the performance indicators of the reagents used.

Seven detection kits from four chemiluminescence systems were used in the current study. All of the kits have been permitted for use via the emergency approval of the China National Drug Administration or the EU CE sales and have been applied to clinical detection. According to the requirements of the People's Republic of China Health Industry Standard WS/T 505–2017 ‘Qualitative Measurement Performance Evaluation Guidelines’,^13^ the performance indicators of qualitative kits should focus on repeatability, clinical accuracy (including clinical sensitivity and specificity) and verification of the cut-off value. The results showed that the repeatability of all detection systems is in line with the manufacturer's statement, but the variance among them is relatively large. Specifically, the coefficients of variation regarding B-IgM and B-IgG are larger than those of the others.

According to the WS/T 494–2017 guideline, the sensitivity and specificity of qualitative items for different occasions are also regulated.^14,15^ In the use of preliminary screening tests, the sensitivity should be greater than 95%. In the case of diagnosis, both the sensitivity and specificity should be greater than 95%. In a confirmed diagnostic test, the specificity should be greater than 98%.^12^ According to the results of the current study, the clinical sensitivity and specificity of all detection systems do not meet the requirements of screening, diagnosis and confirmation of diagnosis experiments. Therefore, all detection systems cannot be used independently for the diagnosis of SARS-CoV-2 infections and need to be used together with nucleic acid tests and clinical symptoms.

Regarding the confounding factors influencing detection results, we divided controls into subgroups that included patients with AIDS, tumours, or pregnancy and older people over 90 years old.^6,16,17^ The results of the current investigation showed that B_IgM has the lowest sensitivity, indicating the possibility of more false negatives that can occur during a period of viral infection or in patients with low immunity.

B_IgG has the lowest specificity, which indicates a higher false-positive rate; this situation is prone to occur in special patients, such as those with AIDS, solid tumours, or pregnancy and elderly individuals.^18^ The reason for false positives may be due to some interfering substances (such as rheumatoid factor, which is homologous to the kit antibodies) present in the specimens. Simultaneously, according to the area under the ROC curve of each detection system, the diagnostic accuracy of B_IgM and B_IgG was also the worst, and the diagnostic accuracy of the other systems was better. In addition, according to the ROC curve and Youden’s index, the best diagnostic thresholds were exhibited by A-IgM and C-IgG, and those of the others were inconsistent with the manufacturer's declaration. The optimal thresholds of A_IgG, B_IgM, C_IgM and D_Ab are less than the cut-off value, indicating increased false-positive results. The optimal threshold of C_IgM is greater than the cut-off value, indicating additional false-negative cases.

Therefore, the laboratory should conduct the necessary performance evaluation of the selected anti-novel coronavirus antibody, carefully interpret the results of the anti-novel coronavirus antibody, perform necessary further testing requirements and reduce missed diagnoses and misdiagnoses.
